# In Vitro and In Vivo Isolation and Characterization of Duvenhage Virus

**DOI:** 10.1371/journal.ppat.1002682

**Published:** 2012-05-24

**Authors:** Penelope Koraka, Byron E. E. Martina, Jouke M. Roose, Pieter-Paul A. M. van Thiel, Geert van Amerongen, Thijs Kuiken, Albert D. M. E. Osterhaus

**Affiliations:** 1 Department of Virology, Erasmus Medical Center, Rotterdam, The Netherlands; 2 Department of Infectious Diseases, Tropical Medicine and AIDS, Academic Medical Center, University of Amsterdam, Amsterdam, The Netherlands; Thomas Jefferson University, United States of America

## Abstract

A fatal human case of Duvenhage virus (DUVV) infection in a Dutch traveller who had returned from Kenya was reported in 2007. She exhibited classical symptoms of rabies encephalitis with distinct pathological findings. In the present study we describe the isolation and characterization of DUVV *in vitro* and its passage in BALB/c mice. The virus proved to be neuroinvasive in both juvenile and adult mice, resulting in about 50% lethality upon peripheral infection. Clinical signs in infected mice were those of classical rabies. However, the distribution of viral antigen expression in the brain differed from that of classical rabies virus infection and neither inclusion bodies nor neuronal necrosis were observed. This is the first study to describe the *in vitro* and *in vivo* isolation and characterization of DUVV.

## Author Summary

Lyssaviruses have been known for centuries to cause lethal encephalitis in animals and humans, representing a serious public health problem especially in developing countries. Little is known about the way that lyssaviruses in general, and Duvenhage virus in particular cause disease. Studies of pathogenesis have been hampered by the fact that the virus has not yet been propagated and characterized extensively. In this paper, we describe the characterization of Duvenhage virus in vitro. Further, we characterized the virus in BALB/c mice. We compared Duvenhage virus with a wild type rabies virus (silver-haired bat rabies virus) and we found that while in vitro the differences of these two viruses were not significant, the in vivo characteristics of these two viruses differed significantly. Histological analyses of infected mouse brains suggest that differences in virulence may be associated with difference in tropism. Elucidating the differences in pathogenesis between different lyssaviruses might help us in the design of novel treatment protocols.

## Introduction

Infection with Duvenhage virus (DUVV) causes lethal encephalitis in humans and animals. Although DUVV infection is prevalent among bats in Africa, reports of human infections are rare and limited to three fatal cases to date, two from South Africa and one from Kenya [1]–[3]. The clinical manifestations of human rabies encephalitis, caused by any of the lyssaviruses, are typically divided into four stages: 1) prodromal phase (local neuropathic reactions at the inoculation site); 2) acute neurological phase (signs of aggression, fear for water and air, fluctuating consciousness, weakness and inspiratory spasms); 3) comatous phase; and 4) death. No effective treatment is available for rabies to date. The prototype virus of the lyssavirus genus; rabies virus (RABV) has a world-wide distribution and is usually transmitted through the bite of a rabid carnivore. Bat species are important reservoirs for RABV in North and South America. Ten additional virus species have been recognized within the Lyssavirus genus, which are mainly carried by bats (with the notable exception of Mokola virus) and are geographically more restricted. African lyssaviruses include Lagos bat virus, (LBV) Mokola virus, (MOKV) and DUVV. European bat lyssaviruses 1 and 2 (EBLV 1 and 2 respectively), Irkut (IRKV), Aravan (ARAV), Khujant (KHUV) and West Caucasian bat virus (WCBV) cause sporadic cases in Europe and Asia. Australian bat lyssavirus (ABLV) is restricted to Australia. DUVV although genetically closely related to RABV, causes different lesions in humans: RABV infection is associated with eosinophilic cytoplasmic inclusion bodies in neurons (Negri bodies) while inflammation is usually not prominent [4], [5]. In contrast, Negri bodies have not been observed in human DUVV infections while extensive inflammation and cell death were found [1], [2]. Similarly, extensive cell death and neuronal damage has been described in human cases of EBLV infection [6]. These differences in lesions suggest differences in the pathogenesis of the infection by the different members of the lyssavirus genus. However, data from human cases of DUVV infections should not be generalized since only three human cases have been described to date. DUVV has not been previously propagated *in vitro* and *in vivo*, hence the limited studies on DUVV pathogenesis. The present study describes the isolation and characterization of the DUVV that caused a fatal infection in a Dutch traveler who had visited Kenya in 2007 [2], [3]. Furthermore we describe passage of the virus in BALB/c mice, thus describing an animal model to further study the pathogenesis of DUVV infection. In the present paper we compare the virulence of DUVV in mice with that of two rabies viruses: the highly pathogenic, wild-type silver-haired bat rabies virus (SHBRV) and the laboratory adapted, attenuated Pasteur rabies virus (RABV-PV). Since different rabies virus isolates vary considerably in their pathogenic potential and our knowledge of DUVV pathogenesis is limited, we chose to compare DUVV-NL07 with two very different rabies virus isolates in order to be able to place DUVV in the spectrum of rabies pathogenesis.

## Results

### Isolation and genome sequence of DUVV-NL07

**Figure 1:**
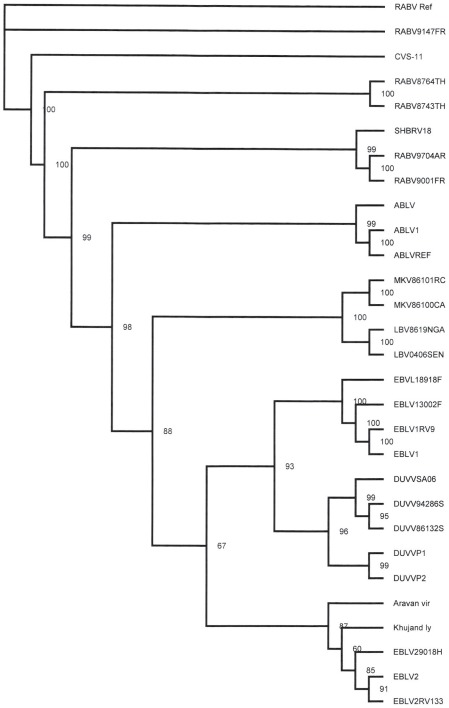
Phylogenetic tree showing the relationship of DUVV-NL07 strain with the different lyssaviruses based on complete genome sequence. Phylogenetic tree (unrooted) was constructed with Tree Puzzle version 5.2 software [31] using the maximum likelihood principle and the Quartet Puzzling algorithm to determine the substitution models that would best fit our data sets. Numbers above the branches indicate the percentage of 10000 puzzling steps. Puzzling support values above the 70% are significant. Accession numbers of all isolates used for the construction of the tree are given in Table S1.

Samples taken for diagnostic purposes from different parts of the human brain [2] were inoculated onto N2a cells and the cultures were followed for 28 days. Virus was isolated from a sample taken from the thalamus as shown by the increase of viral RNA over time in the absence of cytophathic changes. The culture supernatant (primary isolate) was subsequently used for RNA isolation and sequencing of the complete genome of the virus (DUVV-NL07). To confirm that the primary isolate could be propagated in mice, intracranial inoculation of 3-day old BALB/c mice (n = 12) was performed using 100–200 TCID_50_ of the primary isolate of DUVV-NL07, which resulted in 100% mortality within 5 days post infection (DPI). Virus could be recovered from the brain of infected animals and after three *in vivo* passages the virus was still 100% lethal in newborn mice. The complete nucleotide sequence of the primary isolate was deposited in GenBank (accession number JN986749). We next determined the phylogenetic relationship of DUVV-NL07 with other members of the genus *Lyssavirus*. A phylogenetic tree constructed with whole genome sequences of members of all 11 lyssavirus species confirmed that DUVV-NL07 indeed belongs to DUVV species (Figure 1). The DUVV-NL07 isolate proved to be 93% identical on nucleotide level to the other three DUVV isolates from South Africa for which the complete genome sequences are available. The identity between the three South African isolates is up to 99%. The position of DUVV-NL07 outside the South African cluster suggests a genetic variant that circulates in Kenya. When the deduced amino acid sequences of the individual genes were compared with published sequences, the highest identity of DUVV-NL07 with other DUVV isolates was observed in the nucleoprotein and matrix proteins (99% identity with eight published sequences of each gene) followed by the phosphoprotein and the polymerase (96% identity with seven and three published sequences of the respective genes). The glycoprotein G was the most divergent protein (95% identity with six published sequences). An earlier in-frame initiator was seen in the matrix protein resulting in 12 additional amino acids at the N-terminal of matrix protein, similar to what has been described for ABLV [7]. The sequence of the primary DUVV-NL07 isolate was also compared with the partial sequence of the N protein obtained directly from the brain material of the patient [2]. These sequences were 99% identical on the nucleotide level with only three nucleotide differences reported between the sequence deposited from the original brain material and the primary isolate of DUVV-NL07 (position 542: G vs A, position 545: Y vs C, position 551: T vs C).

Virus stocks were prepared by two additional passages of the primary isolate on human neuroblastoma cells (SK-N-SH). The nucleotide sequence of the passaged virus (P3) was 98% identical to the primary isolate. The differences between primary isolate and P3 virus were mainly silent with only one amino acid substitution (a phenylalanine was substituted into a tyrosine in the P3 virus) in the L gene. All subsequent experiments described here were carried out with the primary isolate that had been passaged twice on SK-N-SH cells.

### In vitro and in vivo characteristics of DUVV-NL07

**Figure 2:**
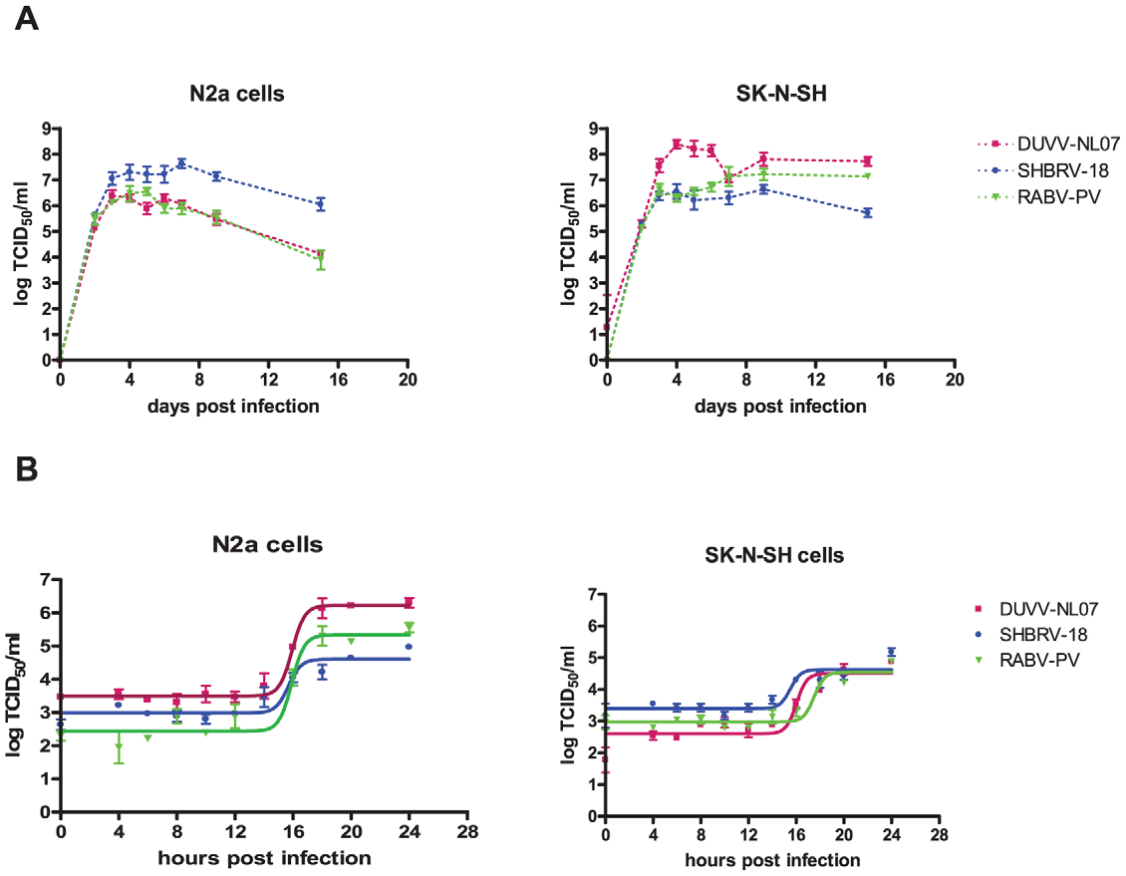
Replication characteristics of DUVV-NL07 in mouse or human neuroblastoma cells. N2a cells (left panels) and SK-N-SH cells (right panels) were inoculated with m.o.i = 0.01 (a) and m.o.i. = 10 (b) Experiments were performed in triplicate and data represent mean ± standard error of the mean (SE). Replication kinetics of DUVV-NL07 (purple) were compared with RABV-PV (green) and SHBRV-18 (blue).

In the focal infection experiment, the replication kinetics of the three different viruses were compared on N2a cells infected at an m.o.i. of 0.01. DUVV-NL07 and RABV-PV showed peak virus titres by day 3 post infection (DPI; Figure 2a), a day earlier compared to SHBRV-18. Infection with any of the three viruses proved to be non-cytopathic in these cells, which remained persistently infected for the 17 days follow up period, although virus titers started to decrease from 7 DPI onwards. In contrast, peak virus titers were measured by 4 DPI in SK-N-SH cells for DUVV-NL07 and SHBRV-18 viruses and by 7 DPI for RABV-PV (Figure 2a). In addition, similar titers were obtained from DUVV-NL07 and RABV-PV in SK-N-SH cells compared to slightly higher titers obtained from SHBRV-18 infected N2a cells. In contrast, infection of SK-N-SH cells with DUVV-NL07 resulted in almost 2log_10_ higher titers compared to the titers obtained from RABV-PV and SHBRV-18 infected cells. To study the short term dynamics of DUVV-NL07 replication, a one-step growth curve was performed on mouse and human neuroblastoma cells at an m.o.i. of 10. The eclipse period for all three viruses was between 14 and 16 hours on N2a cells. Slightly different eclipse periods were found on human neuroblastoma cells for DUVV-NL07 and SHBRV-18 (between 14 and 16 hours) compared to RABV-PV (between 16 and 18 hours) (Figure 2b). At 24 hours, 100% of the cells were infected (Figure S1 and S2) and high titers were detected in the culture supernatant (Figure 2b). Similar titers were found on SK-N-SH cells and N2a cells.

### Neuroinvasion studies and serum antibody development

**Figure 3:**
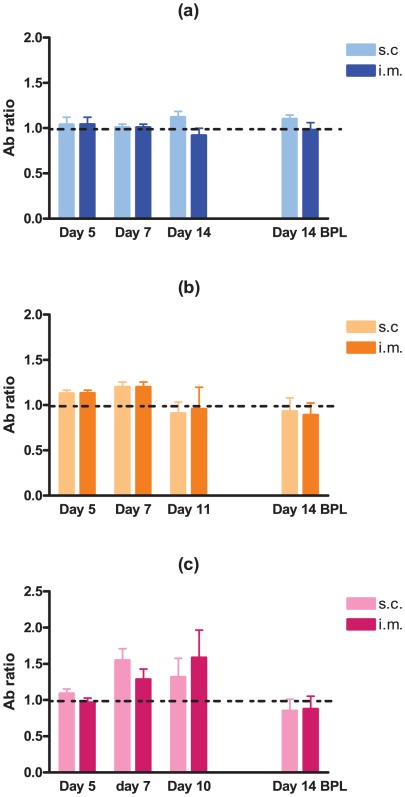
Development of RABV-specific antibodies over time in three- and eight-week old mice. BALB/c mice were inoculated i.m or s.c with (a) 10^2^ TCID_50_, (b) 10^4^ TCID_50_ (c) 10^6^ TCID_50_ of DUVV-NL07 or DUVV-NL07 BPL control. Dotted lines indicate threshold level of the assay.

Several studies have demonstrated that neuro-invasiveness and neurovirulence of RABV are strain-dependent [8]–[10]. We sought to determine the kinetics and minimum dose of DUVV-NL07 that can cause disease upon peripheral inoculation in 3-week and 8-week old BALB/c mice. We found that DUVV-NL07 was neuroinvasive for both 3-week and 8-week old mice inoculated with 10^6^ TCID_50_ or 10^4^ TCID_50_ either i.m or s.c (Table 1). Virus was detected in the brains of animals as early as 7 days post inoculation and clinical signs (including ruffled hair, hunched position and muscle weakness) were apparent from 10 days post inoculation onward. On day 10 post inoculation seven out of 60 inoculated animals developed hind limb paralysis. As shown in Table 1, more animals that received 10^6^ TCID_50_ developed paralysis compared to animals receiving 10^4^ TCID_50_. There was no strong association between development of paralysis and route of inoculation, age or level of viral RNA in the brain. Virus was not detected in the brain samples of animals inoculated with 10^2^ TCID_50_. In addition, no viral RNA was detected in samples taken from the site of inoculation (muscle for i.m. inoculation or skin for s.c. inoculations) and the draining lymph nodes, early after inoculation (3–7 DPI.). In order to assess whether virus replication was necessary to induce antibody response or whether the inoculated antigen burden alone was sufficient to induce antibody response, we compared antibody production in animals inoculated i.m. and s.c. with DUVV-NL07 and BPL-inactivated virus controls. As depicted in Figure 3, on day 7 antibodies could be measured in 30/40 mice inoculated with 10^4^ (Figure 3b) or 10^6^ (Figure 3c) TCID_50_ of DUVV-NL07. On the day that animals had to be euthanized because of development of symptoms (day 11 or 10 post inoculation), antibodies were detected in 33/40 animals which had received 10^4^ (Figure 3b) or 10^6^ (Figure 3c) TCID_50_ of DUVV-NL07 respectively. In animals inoculated with 10^2^ TCID_50_ of DUVV-NL07 no specific antibody response was detected (Figure 3a). Similarly, all animals that received the same antigenic burden of BPL-inactivated virus did not induce specific antibody response (Figure 3a, b, and c). The route of inoculation did not influence significantly the antibody production of animals receiving any dose of DUVV-NL07 at 5, 7 or >10 days post inoculation (P>0.05; Figure 3). The use of RABV-PV antigen in the ELISA could explain the low antibody titers that DUVV-NL07 inoculated mice developed after inoculation with 10^6^ TCID_50_ or 10^4^ TCID_50_.

**Table 1:** In vivo replication of DUVV-NL07.

		8-week old BALB/c			3-week old BALB/c		
		Day 7	Day 10	Total	Day 7	Day 10	Total
10^6^	i.m.	−(0/5)	10^7^ (2/5)^1^	**2/10**	10^7^ (2/5)	10^6^ (3/5)^1^	**5/10**
TCID_50_/mouse	s.c.	10^7^ (2/5)	10^7^ (2/5)^1^	**4/10**	−(0/5)	10^8^ (2/5)^1^	**2/10**
10^4^	i.m.	−(0/5)	10^6^ (3/5)^1^	**3/10**	−(0/5)	10^8^ (1/5)^1^	**1/10**
TCID_50_/mouse	s.c.	−(0/5)	10^8^ (1/5)^1^	**1/10**	10^4^ (1/5)	−(0/5)	**1/10**
10^2^	i.m.	−(0/5)	−(0/5)	**0/10**	−(0/5)	−(0/5)	**0/10**
TCID_50_/mouse	s.c.	−(0/5)	−(0/5)	**0/10**	−(0/5)	−(0/5)	**0/10**

Viral RNA detected in the brain of animals (mean values expressed in copies/ml) infected with high, intermediate or low dose of DUVV-NL07 via peripheral route. The number of DUVV-NL07 RNA positive animals per total number of animals inoculated is indicated within brackets. Numbers in superscript indicate the amount of animals that developed paralysis in the respective groups.

### Virulence studies

**Figure 4:**
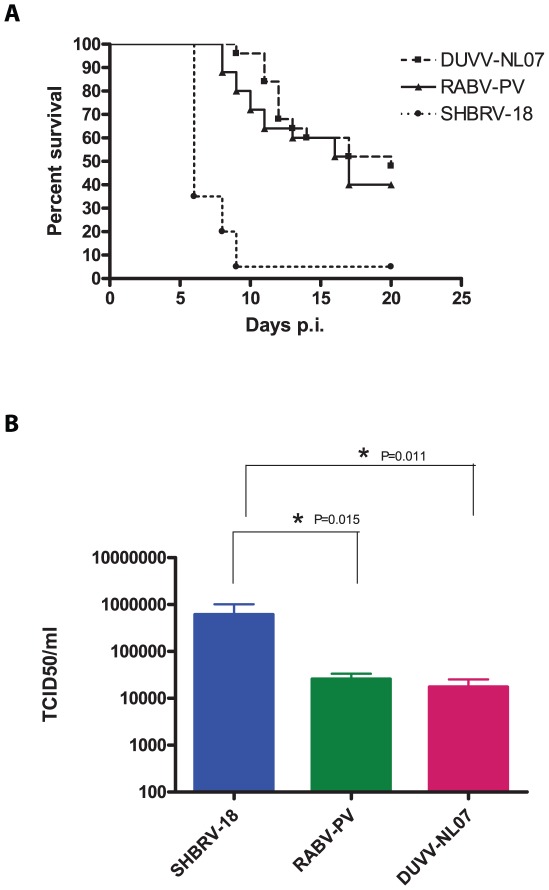
Virulence of lyssaviruses in eight-week old BALB/c mice. (A): survival curves of animals infected with DUVV-NL07 (n = 25), RABV-PV (n = 25) and SHBRV-18 (n = 20). Animals were infected i.m. with 10^6^ TCID_50_ of DUVV-NL07 (squares, dotted line), RABV-PV (triangles, straight line) and SHBRV-18 (circles, dotted line) and followed for clinical signs for 20 days. (B): Infectious viral titres recovered from the brains of mice infected i.m with 10^6^ TCID_50_ of DUVV-NL07, RABV-PV or SHBRV-18. *: statistically different (two-tailed, Mann-Whitney test).

Since the differences in outcome of infection between the respective routes of inoculation and age groups were subtle, we decided to study virulence of DUVV-NL07 in 8-week old mice (n = 25) infected with 10^6^ TCID_50_ virus via the i.m. route. SHBRV-18 and RABV-PV were used as reference controls. Eight-week old mice were chosen for this purpose since they have a fully developed immune system and fully myelinated brain. As shown in Table 2, DUVV-NL07 reached the brain by day 9 after i.m inoculation (as determined by PCR), slightly later than RABV-PV and SHBRV-18 (both on day 5 p.i). Infection with DUVV-NL07 followed a similar course as infection with RABV-PV: clinical manifestations appeared at about the same time (between days 8 and 15 p.i.) and mortality rates were similar (52% and 60% respectively; Figure 4a). Clinical signs were typical of rabies encephalitis including lethargy, ruffled hair, muscle weakness, loss of body-weight, and progressive paralysis of one or both hind-limbs. DUVV-NL07 infected animals developed paralysis in both hind-limbs and became moribund approx. 3 days after onset of clinical signs. RABV-PV infected animals developed paralysis of both hind-limbs by day 3 post onset of clinical signs without becoming moribund. Clinical signs were first observed in SHBRV-18 infected animals on day 5 and infection was more severe resulting in mortality rates up to 95% by day 9 p.i. (Figure 4a). The clinical manifestations of SHBRV-18 infected animals differed significantly from those observed in DUVV-NL07 or RABV-PV infected animals: signs developed very rapidly (within 12 hours post onset of clinical signs animals had to be euthanized) and included aggressive behaviour, myoclonus and torticollis. Hind-limb paralysis was not frequently observed and animals continued to be very active. Next, we compared virus titers in the brain of infected animals at the time of euthanasia. As depicted in Figure 4b, significantly higher virus titers were recovered from animals infected with SHBRV-18 as compared to animals infected with DUVV-NL07 (P = 0.011) or RABV-PV (P = 0.015). No significant differences were observed in the viral titers recovered from brains of DUVV-NL07 and RABV-PV infected animals (P = 0.45).

**Table 2:** Virulence of DUVV-NL07, SHBRV-18 and RABV-PV in 8-week old mice inoculated i.m. with 10^6^ TCID_50_.

		8-week old BALB/C				
		Day 3	Day 5	Day 7	Day of euthanasia^1^	Total
DUVV-NL07	brain	0/5	0/5	0/5	13/25	**13/40**
	muscle	0/5	0/5	0/5	n.d.	**0/15**
RABV-PV	brain	0/5	3/5	2/5	15/25	**30/40**
	muscle	0/5	0/5	0/5	4/5	**4/20**
SHBRV-18	brain	0/5	1/5	n.d.	19/20	**20/30**
	muscle	0/5	0/5	n.d.	5/5	**5/15**

The number of positive animals (viral RNA) per total number of animals tested is indicated.

1 Day of euthanasia varied between the three viruses (day 9–17 for DUVV-NL07, day 8–17 for RABV-PV and day 6–9 for SHBRV-18). See also Figure 4, survival curves.

### Histopathology and immunohistochemistry

**Figure 5:**
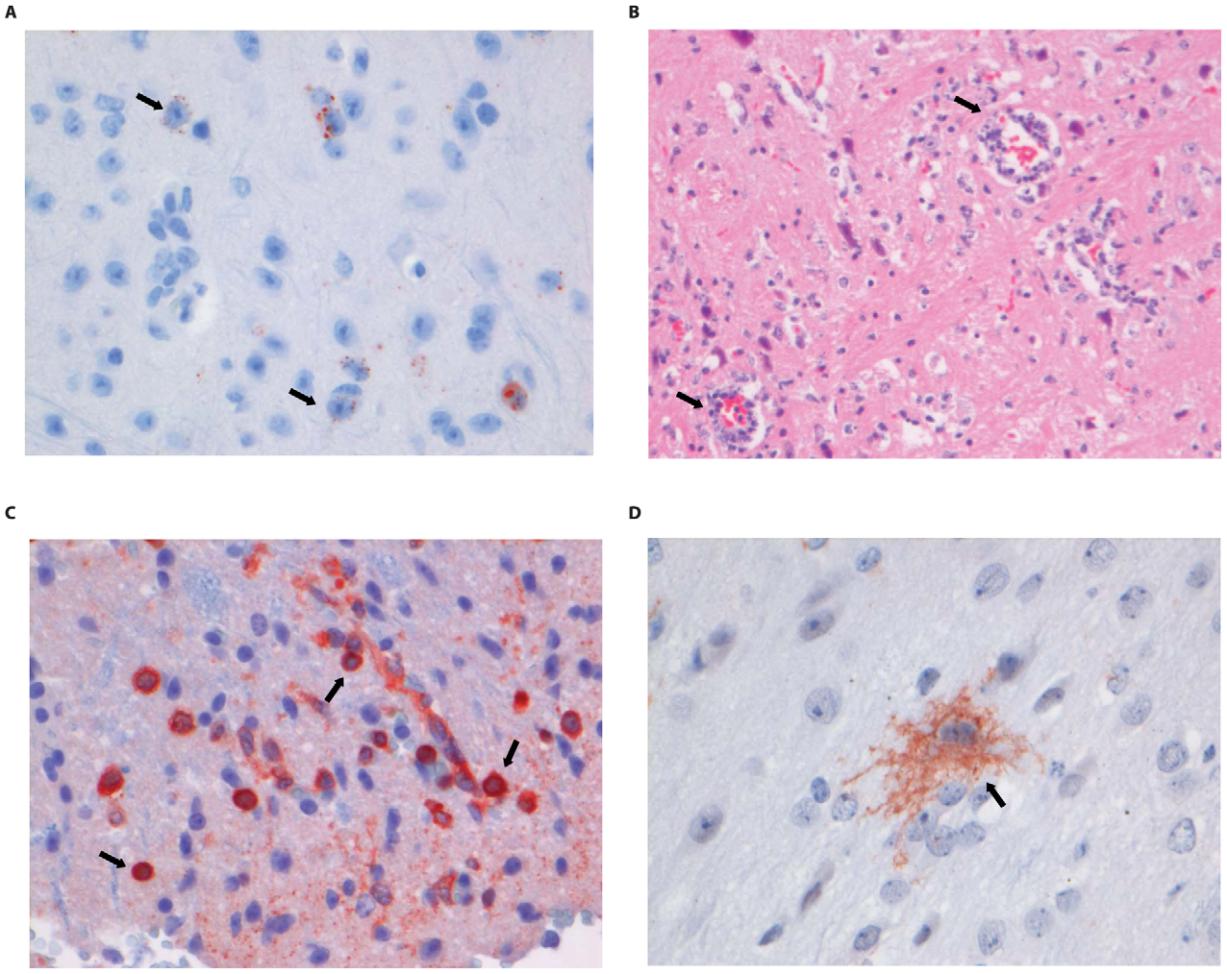
Histopathology of 8-week old BALB/c mice infected i.m. with 10^6^ TCID_50_ of DUVV-NL07. (A) Neo-cortical neurons stained with anti-NP rabies antibody, (B) HE staining of spinal cord section illustrating perivascular cuffing (objective 40×), (C) CD3+ cells infiltrating the neuropil of the spinal cord, (D) activated microglia (Iba1 staining; 40× objective) in the brainstem. Examples of positively stained cells are indicated by block arrows.

**Figure 6:**
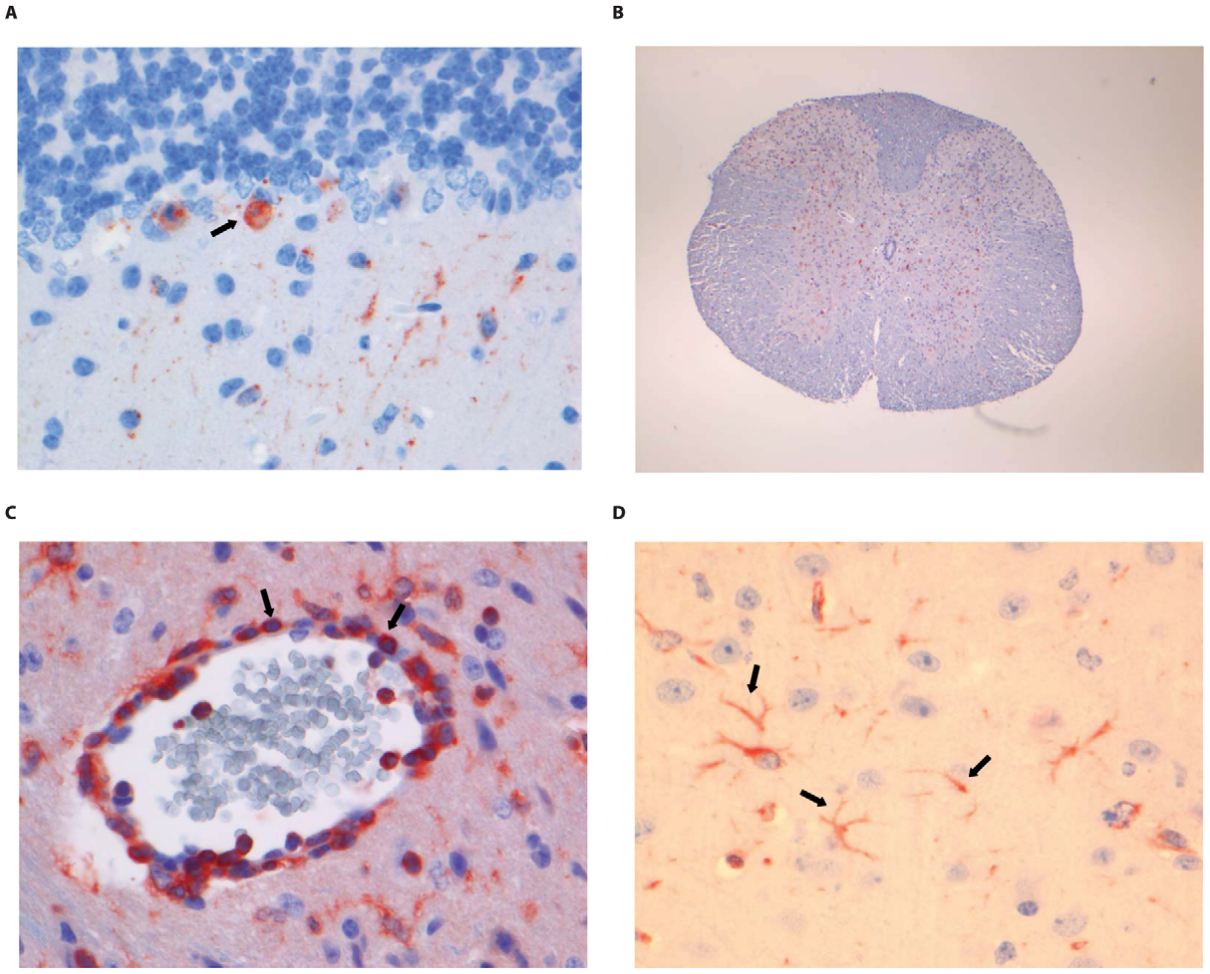
Histopathology of 8-week old BALB/c mice infected i.m. with 10^6^ TCID_50_ of RABV-PV. (A) Purkinje cell layer of the cerebellum stained with anti-NP rabies antibody, (B) Extensive rabies virus antigen expression in the spinal cord (anti-NP rabies staining; 10× objective). (C) CD3+ staining in a perivascular cuff of the spinal cord. (D) astrocytosis in the cerebrum of infected animals (GFAP staining; 40× objective). Examples of positively stained cells are indicated by block arrows.

**Figure 7:**
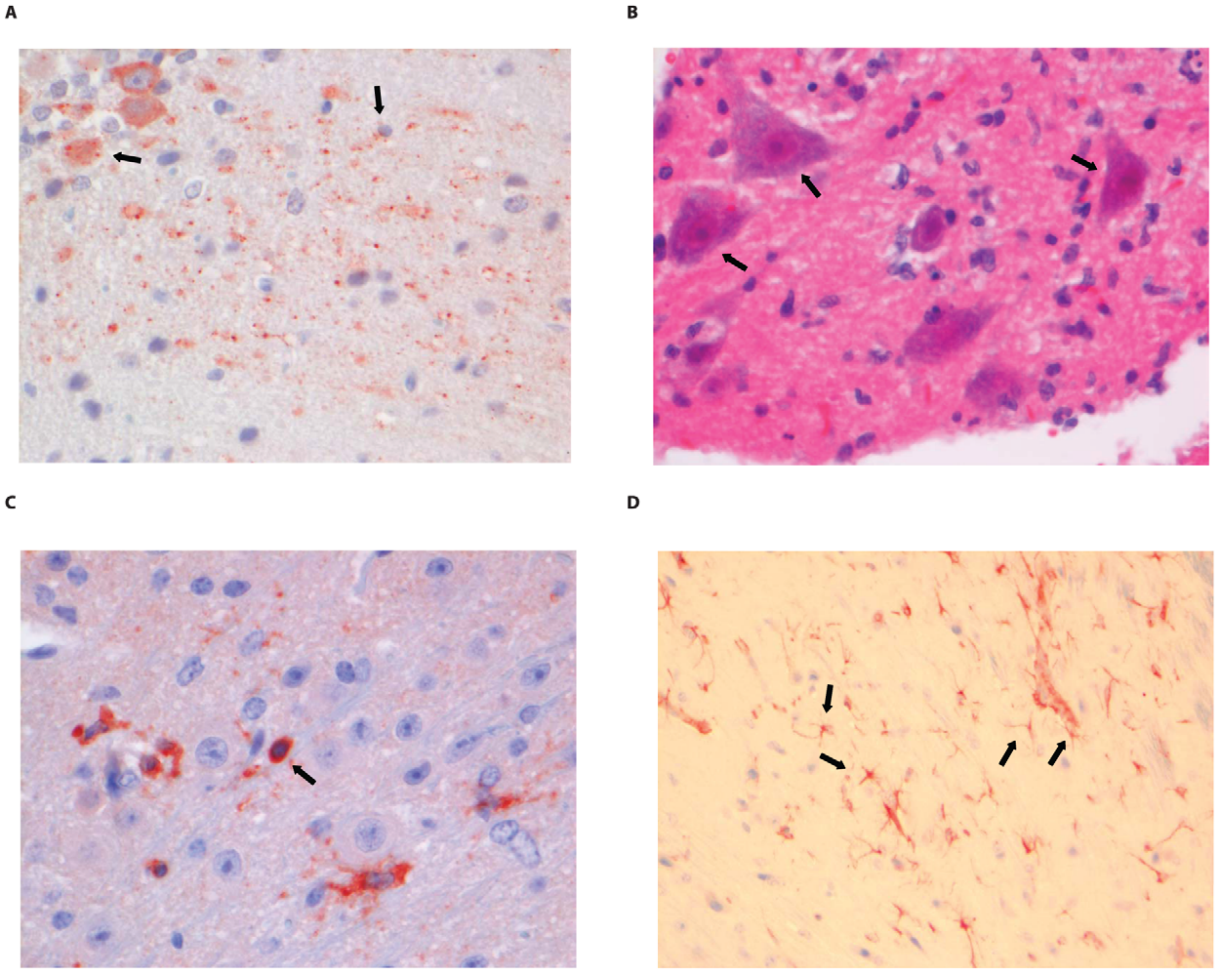
Histopathology of 8-week old BALB/c mice infected i.m. with 10^6^ TCID_50_ of SHBRV-18. (A) The dentate nucleus of the cerebellum stained with anti-NP rabies antibody, (B) necrotic neurons in the spinal cord (HE staining; 40× objective). (C) CD3+ cells infiltrating the neuropil of the spinal cord. (D) astrocytosis in the brainstem of infected animals (GFAP staining; 40× objective). Examples of positively stained cells are indicated by block arrows.

Most wild type RABV strains are known to induce few pathological changes in the brain of infected humans or animals. The pathological changes and tropism of DUVV for brain compartments are unknown. HE and LFB-staining of brains from DUVV-NL07, SHBRV-18 and RABV-PV infected animals showed no evidence of necrosis or demyelination. Staining with anti-RABV-NP antibodies showed extensive antigen expression in infected brains. Antigen expression was seen as characteristic granular staining in the cytoplasm, axons and dendritic processes of infected neurons of moderate (DUVV-NL07; Figure 5A) to strong (RABV-PV; Figure 6A, SHBRV-18; Figure 7A) signal. All three virus infections cumulated in the presence of infected neurons in all parts of the brain: brainstem, cerebellum and cerebrum. However, the pattern of spread differed among the three viruses. DUVV-NL07 infection was first observed in few neurons in the molecular layer of the cerebellum at 9 DPI and spread to the cortex of the cerebrum in the following days (up to 10 DPI). By the time that hind-limb paralysis was observed (9–16 DPI), many neurons were positive in cerebrum, cortex hippocampus and brainstem (and somewhat less in the cerebellum). RABV-PV infection was first observed in a few neurons in the brainstem at 5 DPI, and spread to the cortex of the cerebrum in the following days (7 DPI). By 10 DPI many neurons were positive in all areas of the brainstem and cerebrum, but only in the Purkinje cell layer of the cerebellum. By the time hind-limb paralysis was observed (9 DPI onwards), many neurons were still positive in all areas of brainstem and cerebrum. However, in the cerebellum, positive neurons were found only in the granular layer and not in the Purkinje cell layer. SHBRV-18 infection was first observed in a few neurons in brainstem and cerebrum at 5 DPI and spread to the dentate nucleus of the cerebellum in the following days. By 9 DPI many neurons were positive in all areas of brainstem, cerebrum and cerebellum, including the Purkinje cell layer. At the time of paralysis all three viruses were present in the spinal cord and wide-spread antigen expression was seen in cervical, thoracic and lumbar section of the spinal cord (illustrated for RABV-PV in Figure 6B).

Histopathological changes in the brain consisted of perivascular cuffing by mononuclear cells and increased cellularity in the neuropil, both of which generally colocalized with lyssavirus antigen expression. However, the severity and distribution of these changes differed per virus (Table 3). In DUVV-NL07 infection, perivascular cuffs were 1–2 cells thick and consisted mainly of T cells (CD3 positive). Infiltration of the neuropil by T cells was most prominent in the cerebral cortex where 2 to 4 T cells per high power field (HPF; objective 40×) were observed. Increased numbers of astrocytes (GFAP positive) i.e. mild astrocytosis, was observed in white matter of cerebellum, around perivascular cuffs and in hippocampus but not in brainstem. Increased numbers of activated microglia cells (Iba-1 positive) i.e. microgliosis was observed in the brainstem, grey matter of the cerebrum and cerebellum mainly around infected neurons (Figure 5D). In RABV-PV infection, perivascular cuffs were 1 to 2 cells thick. Infiltration of the neuropil by T cells was minimal, less than one per HPF. Mild astrocytosis was visible in white matter of cerebellum, around perivascular cuffs, in cerebral cortex and in brainstem (Figure 6D). Microgliosis was seen mainly in cerebrum and brainstem and somewhat less in the cerebellum (around the Purkinje cell layer). In SHBRV-18 infection, perivascular cuffs were more than two cells thick and consisted of many T cells. Infiltration of the neuropil by T cells was most prominent in the brainstem, where 2 to 3 cells per HFP were observed. Astrocytosis was visible in cerebral cortex, cerebral nuclei and brain stem but not in the cerebellum (Figure 7D). Microgliosis was seen mainly around perivascular cuffs and not in the neuropil. Neuronal necrosis was not seen in the brains of any of the infected mice, irrespective of the infecting virus. Although neither visible by routine HE staining nor with Seller's staining (Figure S4), we did observe inclusion bodies reminiscent of Negri bodies when staining for antigen expression with IHC.

**Table 3:** Overview of histopathological changes observed in the brain and spinal cord of animals infected with DUVV-NL07, RABV-PV or SHBRV-18 virus at the time of paralysis.

	DUVV-NL07	RABV-PV	SHBRV-18
perivascular cuffs (brain)	1–2 CD3+	<1 CD3+	>2 CD3+
perivascular cuffs (spinal cord)	1–2 CD3+	<1 CD3+	>2 CD3+
Cells infiltrating neuropil	2–4 CD3+ (cortex)	<1 CD3+	2–3 CD3+ (brainstem)
	>2 CD3+ (spinal cord)	1–2 CD3+ (spinal cord)	1 CD3+ (spinal cord)
astrocytosis	Cerebellum	Cerebrum	Cerebrum
(GFAP+)	Brainstem	Brainstem	Brainstem
	Perivascular cuffs	Perivascular cuffs	
	Spinal cord	Spinal cord	Spinal cord
microgliosis	Cerebellum	Cerebellum	
(Iba1+)	Cerebrum	Cerebrum	
	Brainstem	Brainstem	
			Perivascular cuffs
	Spinal cord	Spinal cord	Spinal cord

Sections were stained with antibodies against rabies virus nucleoprotein, CD3 (T cells) GFAP (activated astrocytes) and Iba1 (activated microglial cells) as described in the materials and methods. Numbers indicate numbers of positive cells per high power field (objective 40×) when complete brain or spinal cord sections were screened.

In the spinal cords of infected animals acute neuronal necrosis was seen after infection with all three viruses (illustrated for SHBRV-18 in Figure 7B). Perivascular cuffing and mild infiltration of T cells indicated acute inflammation. In DUVV-NL07 infection, perivascular cuffs were 1 cell layer thick and consisted mainly of T cells (CD3 positive). Infiltration of the neuropil by T cells was prominent in cervical, thoracic and lumbar sections (Figure 5C) with more than 2 positive cells per HPF. Astrocytosis and microgliosis were seen mainly in lumbar and thoracic sections of the spinal cord. In RABV-PV infection, perivascular cuffs were 1 cell layer thick and consisted mainly of T cells (Figure 6C). Infiltration of the neuropil by T cells was moderate with 1 to 2 cells per HPF and astrocytosis and microgliosis was mainly seen in thoracic and lumbar sections. In SHBRV-18 infection, perivascular cuffs were 1 cell layer thick consisting mainly of T cells and neutrophils (Figure 7C). Infiltration of the neuropil was minimal with less than 1 cell per HPF and astrocytosis and microgliosis were seen mainly in the thoracic sections of the spinal cord.

## Discussion

Here we describe the *in vitro* isolation of DUVV from the brain of a human patient [3] and its subsequent molecular and biological characterization. DUVV infection in humans is rare and restricted to Africa, with only three cases reported so far. Phylogenetic analysis of lyssaviruses has been largely based on the complete genome or the N gene [11]–[15]. The DUVV-NL07 strain described in this study clusters differently from the bat and human DUVV's reported so far (Figure 1) [1]. At the time of analysis there were only three complete genome sequences and eight N gene sequences available from other DUVV isolates. Although the variation among DUVV strains (including the DUVV-NL07 isolate) is low compared to other lyssavirus species [11], [16], DUVV-NL07 is the most divergent among DUVV isolates both on the nucleotide and amino acid level. Further studies are needed to elucidate the biological relevance of the observed differences between DUVV isolates as has been proposed for different RABV isolates [17], [18].

The one-step growth curve experiment did not reveal any significant differences in replication kinetics between the different viruses, neither on mouse or human neuroblastoma cells. Therefore, the replication kinetics are not an in vitro correlate of virulence. However, we cannot exclude that different amounts of defective interfering particles in the different virus stocks may have influenced the replication kinetics of these viruses. In order to study the spreading potential of the respective viruses, focal experiments were performed. Reduced virus titers were observed in mouse neuroblastoma cell lines as compared to human cell lines. The mechanisms underlying these differences are not clear. It is possible that the two different cell lines differ in viral entry-receptor density or qualitative or quantitative differences in anti-viral response to infection. Alternatively, differences in the rate of cell death due to prolonged culture could also explain the differences observed in the spreading potential of the three viruses in human and mouse cells.

In the case report of van Thiel et al. [2] the crude brain material from the patient did not cause disease in outbred mice with the mouse inoculation test. Consistently, virus or RNA could not be detected in inoculated animals. Similarly, virus was not isolated from newborn inbred mice (BALB/c and C57/Bl6) inoculated with the same crude brain material (data not shown). Even though we could not determine the infectious virus titer in the brain material of the patient by endpoint dilution, the quantitative PCR data suggest that the brain sample used for inoculation of mice contained approximately 10 TCID_50_/ml of infectious virus. Our data confirm the low sensitivity of the mouse inoculation test [19], [20]. Therefore, prior in vitro propagation of the virus proved to be necessary to further study the characteristics of the virus. We have shown that DUVV-NL07 is pathogenic for BALB/c mice upon i.c. and peripheral inoculation. DUVV-NL07 replicated well in mouse and human neuroblastoma cells as well as in hamster fibroblasts (BHK-21-C13, data not shown). Further, the virus proved to be both neuroinvasive and neurovirulent in juvenile and adult BALB/c mice upon infection via the i.m or s.c routes. It was previously shown that neuroinvasiveness of a bat-derived RABV was dependent on the route of the inoculation [17]. The authors speculated that the increased neuroinvasion observed in their study was likely a reflection of the peripheral cell tropism. It is plausible that bat-derived lyssaviruses, such as DUVV-NL07 could replicate better in sites such as the dermis or the subcutaneous space compared to muscles since the natural route of infection via bat scratches would not implicate direct inoculation of the virus into the muscle. In our studies we did not see differences in neuroinvasion between the two different routes of inoculation, neither could we detect replicating virus at the site of inoculation early after infection. This suggests that neuroinvasion might not be dependent on abundant peripheral virus replication. Given the nature of transmission via usually superficial bat-scratches, as was the case in the human DUVV-NL07 infection, it is generally believed that bat-derived lyssaviruses are neuroinvasive at low doses. Indeed, peripheral infection with as little as 10^4^ TCID_50_ of DUVV-NL07 caused encephalitis in a substantial number of mice. Further comparisons revealed that neither route of infection, nor age of the mice led to marked differences in the clinical outcome, antibody response or amount of viral RNA recovered from the brain.

Comparison of in vitro characteristics and in vivo neuroinvasive characteristics and virulence of DUVV-NL07 (after peripheral inoculation) with those of RABV-PV (a mouse adapted strain of RABV) and SHBRV-18, a wild-type highly pathogenic bat-derived RABV strain that had been recovered from a human rabies case [17], showed clear similarities with RABV-PV. In contrast, SHBRV-18 infection exhibited a different tropism for neuronal cells, different clinical manifestations and nearly 100% lethality. These data indicate that DUVV-NL07 has intermediate neuroinvasiveness and virulence while exhibiting characteristics of some of the fixed RABV strains [21]. Similar to RABV-PV and SHBRV-18, the major pathological change observed after DUVV-NL07 infections was perivascular cuffing, whereas no neuronal necrosis (in the brain) was observed. Different lines of research in mouse models have indicated that several aspects of RABV pathogenesis are largely strain dependent, such as activation of the immune system, infiltration of leukocytes into infected brains, gene expression patterns and cell death (reviewed by Fu and Jackson [22]). Some studies have suggested that level of expression of G protein inversely correlates with pathogenicity [8], [23]. However, more recent data suggest that G protein expression levels are not critical for pathogenicity [24] and that it is the capacity of the G protein to promote survival or death signals in the infected neurons that contributes to pathogenicity [25]. It remains to be seen if the G protein of DUVV-NL07 would have the capacity to induce such signals and confer a pathogenic or attenuated phenotype.

Our findings are in agreement with previous studies suggesting that inflammation is not a significant determinant of pathogenesis of DUVV-NL07 or bat-derived RABV [10], [23]. Also tropism of lyssaviruses for different brain compartments has been shown to be largely strain dependent [4], [26], [27]. The mouse cerebellum did not seem to be an important site of replication for DUVV-NL07, since only few cells stained positive for NP-antigen at all time points analysed after infection. The infection appeared largely localized in certain areas of the cerebral cortex and hippocampus even in mice that had reached advanced stages of paralysis. In contrast, both RABV-PV and SHBRV-18 showed a clearly different pattern of spread into the CNS, with Purkinje cells and the dentate nucleus in the cerebellum being the primary site of replication and more than 70% of neurons throughout the brain being infected at the time of euthanasia. Interestingly these histopathological findings largely paralleled the differences in clinical signs observed between infections with the respective virus strains. Overall, all animals exhibited signs of aggression reflecting infection of the cerebrum by the three viruses. Extensive infection of the cerebellum by RABV-PV and SHBRV-18 may have contributed to paralysis whereas infection of the dentate nucleus by SHBRV-18 correlated with the characteristic signs of torticollis. Given the differential tropism patterns of the three viruses for neurons in the cerebellum, it would be interesting to compare the receptor usage of DUVV-NL07 with RABV-PV and SHBRV-18.

Despite the differences seen in the brain, the pathology of the spinal cord of the infected animals seemed similar between the three viruses. Inflammation and necrosis were of similar extent between the three viruses and antigen expression was wide-spread in all sections of the spinal cord. These findings suggest that the differences in pathogenesis of the three viruses are most likely linked to the brain.

In the three human cases of DUVV infection documented so far, the clinical manifestations did not differ from those of classical rabies. We therefore speculate that the severity of clinical outcome seen in rabies is probably attributable to infection or dysfunction of (yet unidentified) highly specialized compartments of the brain, common to all lyssaviruses. It should be noted that the original sequence of the virus from crude brain material was not available. However, the sequence of the primary isolate was 98% identical (on the nucleotide level) and only one amino acid substitution with the P3 virus used for the in vitro and in vivo studies described in this paper. Studies using a molecular clone of DUVV would be needed to further determine the significance of the single amino acid substitution in the L gene after in vitro passage of DUVV. It would be interesting to evaluate the implications of such differences in patient management and identify treatment protocols.

Both the *in vitro* and *in vivo* systems for DUVV-NL07 propagation described in the present study will be valuable tools to further elucidate the pathogenesis of infections with bat lyssaviruses and possible treatment options.

## Materials and Methods

### Ethics statement

All animal experiments described in this paper have been conducted according to Dutch guidelines for animal experimentation and approved by the Animal Welfare Committee of the Erasmus Medical Centre, Rotterdam, The Netherlands. All efforts were made to minimize animal suffering.

### Cells and viruses

Mouse neuroblastoma (N2a) cells and baby hamster kidney (BHK-21 clone 13), a kind gift from Dr. F. Cliquet, (AFSSA, Nancy France), were grown in DMEM supplemented with 5% heat inactivated fetal bovine serum (HI-FBS) and GMEM supplemented with 10% HI- FBS, respectively. Human neuroblastoma cells (SK-N-SH; a kind gift from Dr. C. Prenaud; Institute Pasteur, Paris France) were cultured in DMEM with Glutamax supplemented with 10% HI-FBS. All media were supplemented with antibiotics (100 U penicillin, 100 µg/ml streptomycin) and 2 mM L-glutamine. Cell culture reagents were obtained from LONZA (Lonza Benelux BV, Breda, The Netherlands). All cell lines tested negative for mycoplasma.

The SHBRV-18 (a kind gift of Dr. B Dietzschold, Tomas Jefferson University, Philadelphia, USA), RABV strain Pasteur (RABV-PV), and DUVV isolated from a Dutch patient (DUVV-NL07, described in this study) were grown to high titres on SK-N-SH cells. SHBRV-18 was originally isolated from the brain of an infected human and subsequently passaged *in vivo* and *in vitro* to obtain variant SHBRV-18 [18]. The passage history of RABV-PV has not been documented adequately. Virus titrations were performed in BHK-21-C13 cells as previously described [28] and titres were calculated with the Kärber-Kaplan method [29]. Viruses were inactivated with beta-propiolactone (BPL). To this end, BPL was introduced to a final dilution of 1∶4000 (v/v) and incubated overnight at 4°C to ensure complete viral inactivation. Subsequently, BPL was inactivated for 1 h at 37°C. Inactivated viruses did not replicate on BHK-21-C13 cells in virus titration assays.

### Sequencing of DUVV-NL07 isolate

RNA was isolated from the primary DUVV-NL07 isolate with the Qiagen RNeasy mini kit according to the instructions of the manufacturer (Qiagen Benelux, Venlo, The Netherlands). cDNA was synthesized using random hexamer primers (Invitrogen, Breda, The Netherlands) or oligo dT primer (Invitrogen) and superscript III RT enzyme (Invitrogen) according to the instructions of the manufacturer. Twelve sets of primers spanning the complete genome sequence of RABV-PV were designed in areas conserved with other members of plylogroup I lyssaviruses including the previously known DUVV sequences [14]. Primers were designed using the Primer select module of DNASTAR software (DNASTAR, Madison WI, USA) and adjusted manually to obtain highest identity with the known DUVV sequences. Primer sequences are available from the authors upon request. cDNA was amplified using Taq DNA polymerase (Invitrogen) and DNA fragments were purified from gel and cloned into the pCR4-TOPO vector (Invitrogen). Colonies were sequenced using M13 primers in an ABI3130XL sequencer. Sequences were analysed using the SeqMan module of DNASTAR software and aligned so that the complete DUVV-NL07 genome was obtained from the consensus sequence of at least five colonies.

### Detection and quantification of viral RNA

Real-time PCR for the detection of viral RNA was done using the Taq-Gold TaqMan kit (Applied Biosystems, Nieuwerkerk aan den Ijssel, The Netherlands) and primers/probe combinations previously described [2], [30]. RNA copy numbers were quantified using a standard curve of in-vitro transcribed RNA of known quantities. The detection limit of the qPCR used to determine virus load in the brains of infected animals was 1000 copies/ml.

### Replication kinetics of DUVV-NL07, RABV-PV and SHBRV-18 viruses

The replication kinetics of DUVV-NL07, RABV-PV, and SHBRV-18 were studied in vitro using two type of experiments: a one-step growth curve experiment in which all cells were infected at high m.o.i., and a focal-infection experiment using a low m.o.i. To this end, mouse or human neuroblastoma cells were inoculated in suspension with either of the three viruses at an m.o.i. of 0.01 (focal infection) or m.o.i. of 10 (one step growth curve) for one hour at 37°C. Cells were washed five times with serum free medium, resuspended in growth medium, seeded in plates and incubated at 37°C (T = 0). Supernatant samples were collected in triplicate at the indicated time points, centrifuged at 1000 rpm for 5 min to remove cell debris and stored in −80°C until determination of virus titers on BHK-21-C13 cells.

### Detection of lyssavirus specific antibodies in mouse serum samples

High-binding COSTAR 96-well ELISA plates (Sigma Aldrich, St. Luis, MO, USA) were coated overnight at 4°C with BPL-inactivated RABV-PV antigen. Plates were washed four times with PBS containing 0.05% Tween-20 (PBS-T) to remove unbound antigen and blocked for one hour at 37°C with PBS containing 0.1% (w/v) bovine serum albumin (Sigma) and 0.2% (w/v) skim milk powder (ELK Campina, Eindhoven, The Netherlands) (ELISA buffer). After a washing step, plates were incubated for one hour at 37°C with mouse serum samples serially diluted (2-fold) in ELISA buffer. After removal of unbound antibodies with a washing step plates were incubated with a rabbit anti-mouse IgG HRPO conjugate (DAKO, Glosturp, Denmark) for one hour at 37°C. After a final washing step plates were developed with tetra-methyl-benzidine substrate for 10 min at RT and the reaction was stopped by adding 0.5 N of sulphuric acid. Absorbance was measured at 450 nm with an Infinite F200 TECAN instrument (TECAN Benelux, Giessen, The Netherlands). Antibody ratio was calculated as O.D. sample/cutoff (where cutoff: mean O.D. of the negative controls+3× the standard deviation).

### Animal studies

Neuroinvasive characteristics and virulence of DUVV-NL07 was studied in newborn (3 days old), 3-week or 8-week old BALB/c mice. Newborn mice were euthanized when they reached humane end-points (both hind-limb paralysis). Three-week or 8-week old mice were euthanized on day 3, 5, 7, 10 (n = 5 for each time point) and on the humane end-point (n = 25). Mice were anesthetized with isoflurane prior to inoculation via the indicated route (intracranial; i.c. intramuscular; i.m. or subcutaneous; s.c). Animals were euthanized under anaesthesia by cervical dislocation at the indicated time points and samples were collected immediately for further processing. Brain samples were collected for immunohistochemistry, virus isolation and quantification whereas blood was collected for determination of antibody levels. Eight-week old BALB/c mice were inoculated i.m. with RABV-PV or SHBRV-18 and euthanized on day 3, 5, 7 (n = 5 for each time point) and at the time of the humane end point (n = 25 for RABV-PV and n = 20 for SHBRV-18). Samples were collected as described for DUVV-NL07. Animals were housed in cages of 5 animals per cage, had 12-hour day-night cycle and had constant access to food and water. All animal experiments were approved by the Animal Ethics Committee of Erasmus MC, The Netherlands.

### Histology and immunohistochemistry

Brains and spinal cords were removed and fixed in 10% neutral-buffered formalin, embedded in paraffin and sectioned at 4 µm. Spinal cords were divided in parts of approx. 1 cm thick to obtain cervical, thoracic and lumbar sections. Slides were stained with hematoxylin and eosin (HE) and Luxor Fast blue (LFB) for analysis by light microscopy. Immunohistochemical analysis for virus nucleoprotein and cell-type markers was performed on brain and spinal cord sections using the streptavidin-biotin-peroxidase technique. Briefly, sections were deparaffinized in xylane, re-hydrated in descending concentrations of ethanol and incubated for 10 min in 3% H_2_O_2_ diluted in PBS to block endogenous peroxidase activity. Antigen exposure was performed by incubation for 15 min at 121°C in citrate buffer (0.01 M, pH 6.0). Primary antibodies included goat anti-rabies NP antibody (1∶500 Rabies polyclonal DFA Reagent; CHEMICON), anti-mouse CD3 (T cell marker; 1∶1000 DAKO), rabbit anti-Iba1 (microglial marker, 1∶500; WAKO) and rabbit anti-GFAP (astrocyte marker, 1∶500; ZYMED). A streptavidin-biotin-peroxidase kit (UltraVision Large Volume Detection System Anti-polyvalent, HRP Lab Vision, USA) was used as secondary antibody (goat anti-polyvalent/streptavidin enzyme complex) and 3-amino-9-ethyl carbazole (AEC, Sigma) was used as a substrate. Sections were counterstained with Mayer's hematoxylin and mounted with Kaiser's glycerin-gelatin. Sections incubated without the primary antibody (omission control), isotype controls and use of negative brain tissue confirmed specificity of staining. Sections from animals inoculated with RABV-PV were considered as positive controls. One complete brain section from all infected animals (as demonstrated by positive viral antigen staining) was screened at high power field (objective 40×, approximately 30 high power fields/cerebellum, 30 high power fields/cerebrum and 30 high power fields/brainstem) for determination of CD3, GFAP and Iba1 positive cells. One cervical, one thoracic and one lumbar section of the spinal cord of nine representative animals (n = 3 for DUVV, n = 3 for RABV-PV and n = 3 for SHBRV-18) were screened at high power field as described for the brain sections (approximately eight high power fields per spinal cord section).

### Statistics

Survival curves were made with the Kaplan-Meier method and analyzed with a two-tailed logrank test (Graph Pad version 4). Viral and antibody titers were compared with a two-tailed, non-parametric Mann-Whitney test (Graph Pad version 4).

## Supporting Information

Figure S1 Amount of infected mouse neuroblastoma cells 24 hours after infection. N2a cells were infected (m.o.i. = 10) with (a) DUVV-NL07, (b) RABV-PV and (c) SHBRV-18 and stained with anti-NP FITC antibody.
(PDF)

Figure S2 Amount of infected human neuroblastoma cells 24 hours after infection. SK-N-SH cells were infected (m.o.i. = 10) with (a) DUVV-NL07, (b) RABV-PV and (c) SHBRV-18 and stained with anti-NP FITC antibody.
(PDF)

Figure S3 Amount of infected human neuroblastoma cells seven days after infection. SK-N-SH cells were infected (m.o.i. = 0.01) with (a) DUVV-NL07, (b) RABV-PV and (c) SHBRV-18 and stained with anti-NP FITC antibody.
(PDF)

Figure S4 Seller's staining of brain sections from 8-week old mice. BALB/C mice were infected i.m. with 10^6^ TCID_50_ of DUVV-NL07 (S4a) or RABV-PV (S4b) or SHBRV-18 (S4c). Seller's staining was performed as described in “Laboratory Techniques in rabies” Forth Edition, World Health Organization, Geneva 1996.
(PDF)

Table S1 Virus isolates and their respective accession numbers used to construct the phylogenetic tree depicted in Figure 1.
(DOC)
