# Supplementary material for: In Vitro and In Vivo Isolation and Characterization of Duvenhage Virus
Source: PLoS Pathog. 2012 May 24;8(5):e1002682. doi: 10.1371/journal.ppat.1002682 (PMC3359985; doi:10.1371/journal.ppat.1002682)

Supplementary Figure 2: Amount of infected human neuroblastoma cells 24 hours after infection. SK-N-SH cells were infected (m.o.i.=10) with (a) DUVV-NL07, (b) RABV-PV and (c) SHBRV-18 and stained with anti-NP FITC antibody.

Figure S2a

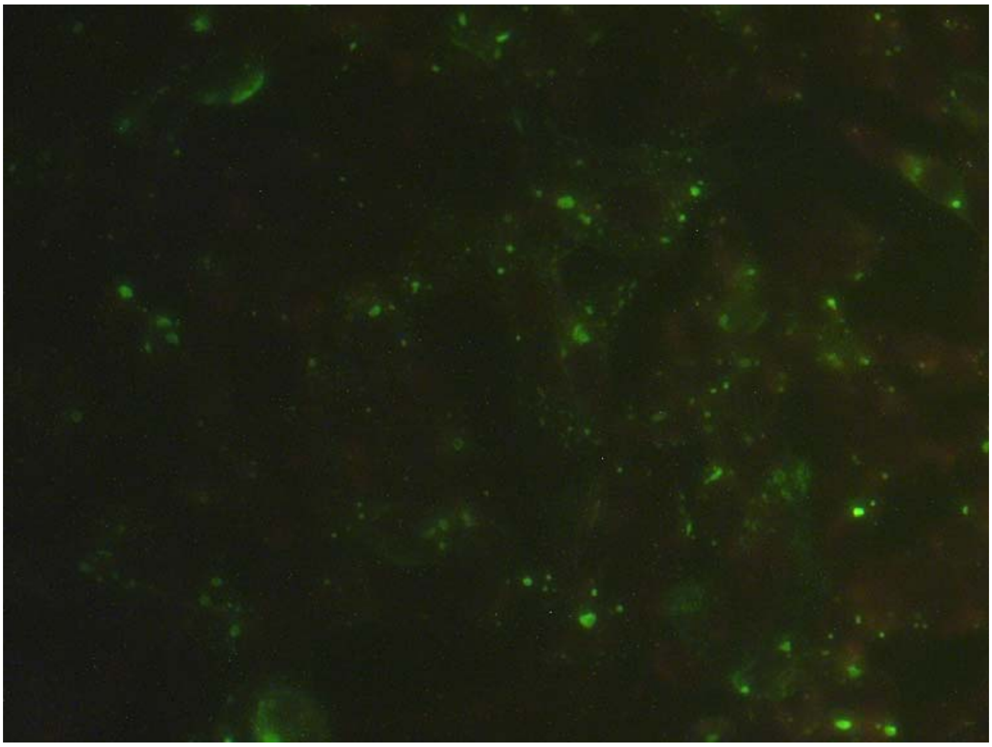

Figure S2b

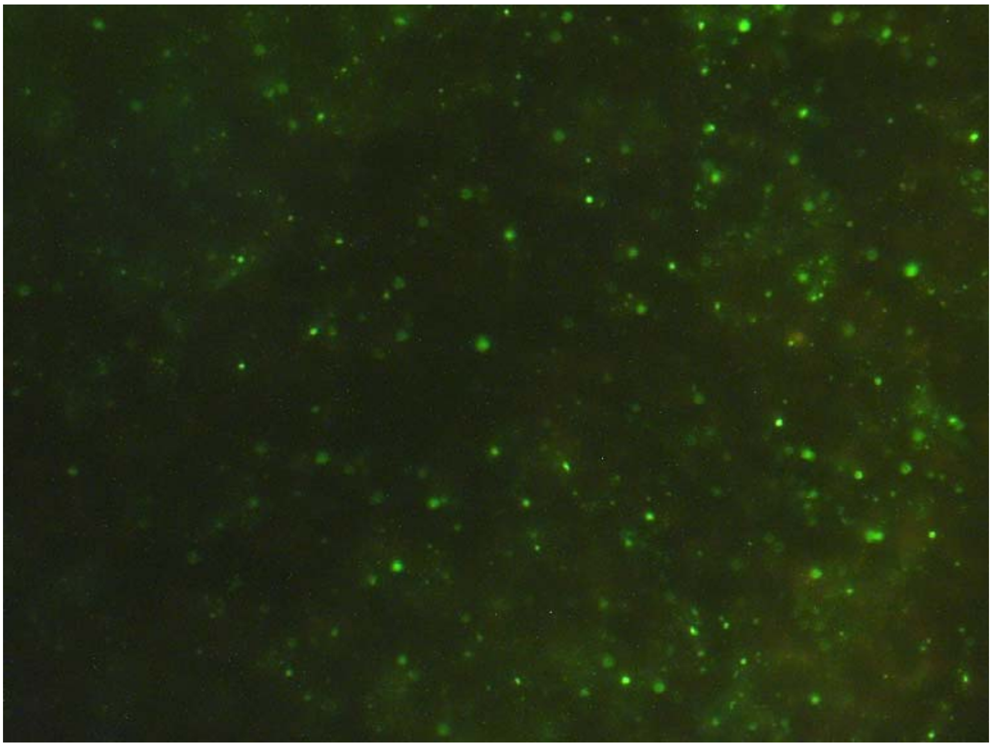

Figure S2c

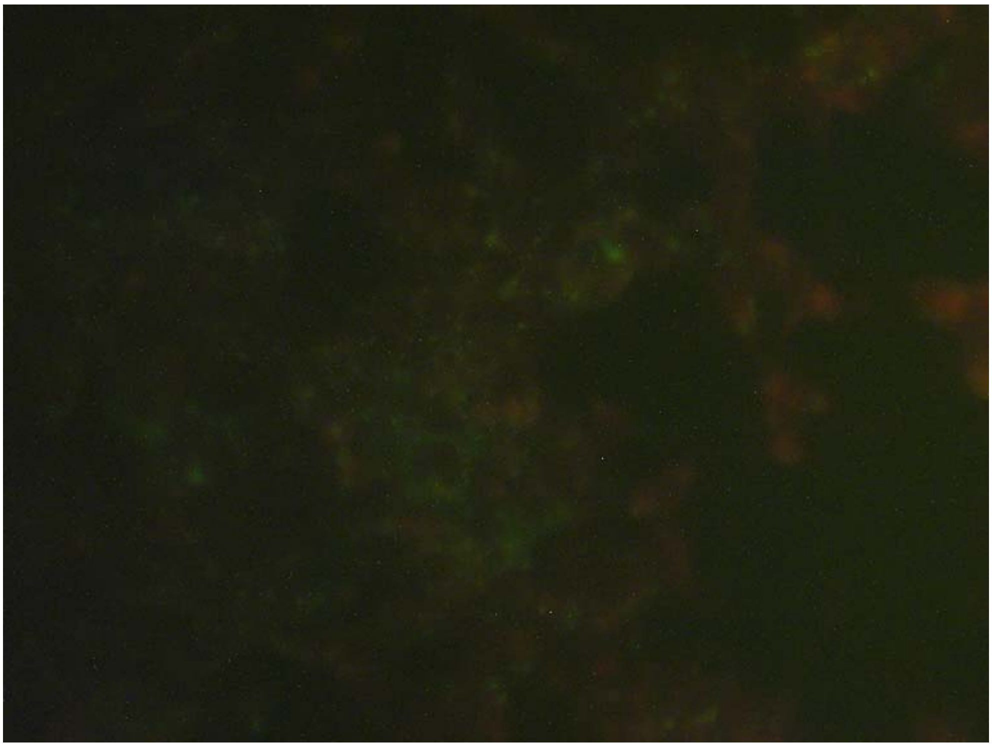

Supplement: Figure S2 — Amount of infected human neuroblastoma cells 24 hours after infection. SK-N-SH cells were infected (m.o.i. = 10) with (a) DUVV-NL07, (b) RABV-PV and (c) SHBRV-18 and stained with anti-NP FITC antibody. (PDF) [file ppat.1002682.s002.pdf]
