# Supplementary material for: In Vitro and In Vivo Isolation and Characterization of Duvenhage Virus
Source: PLoS Pathog. 2012 May 24;8(5):e1002682. doi: 10.1371/journal.ppat.1002682 (PMC3359985; doi:10.1371/journal.ppat.1002682)

Supplementary Figure S4: Seller's staining of brain sections from 8-week old mice. BALB/C mice were infected i.m. with 10^6 $TCID_{50}$ of DUVV-NL07 (S4a) or RABV-PV (S4b) or SHBRV-18 (S4c). Seller's staining was performed as described in "Laboratory Techniques in rabies" Forth Edition, World Health Organization, Geneva 1996.

Figure S4a

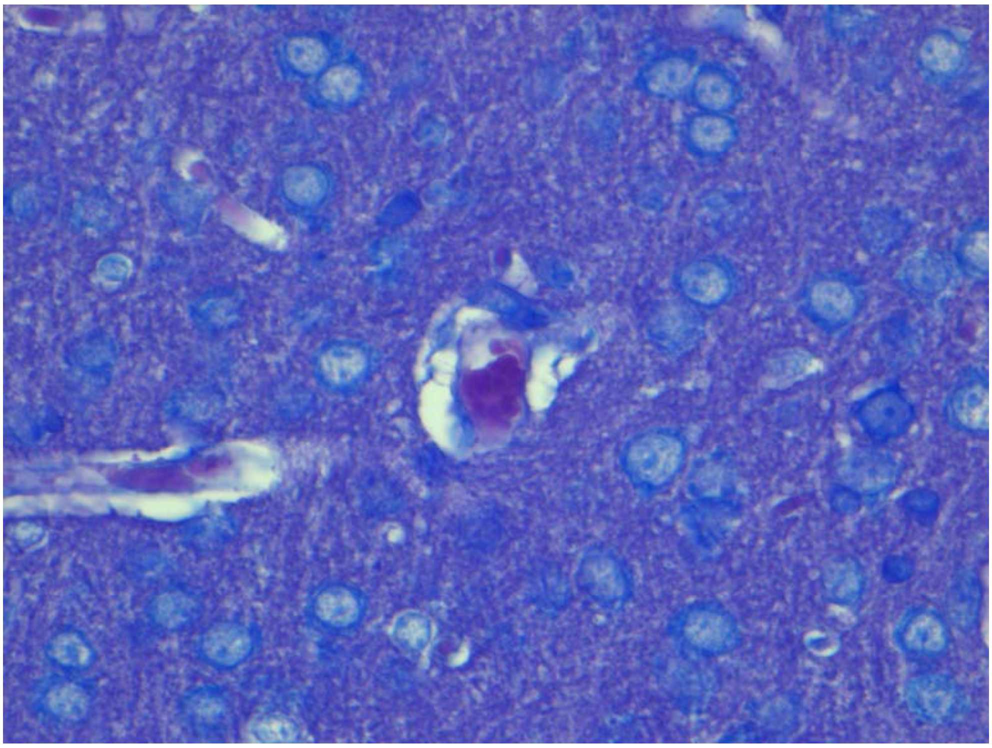

Figure S4b

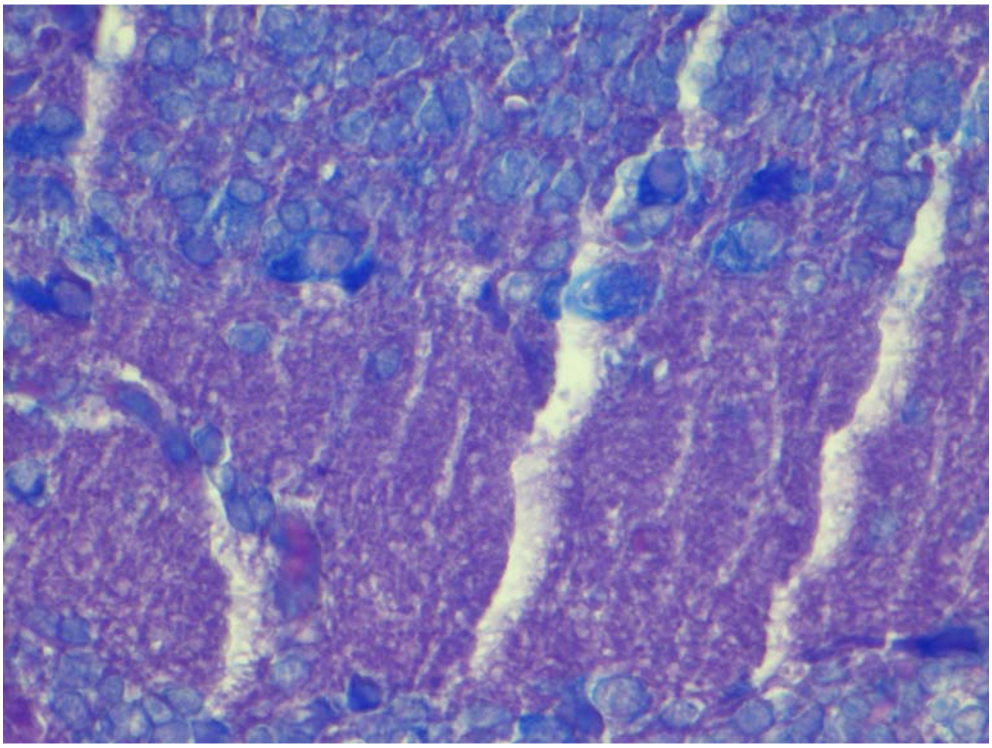

Figure S4c

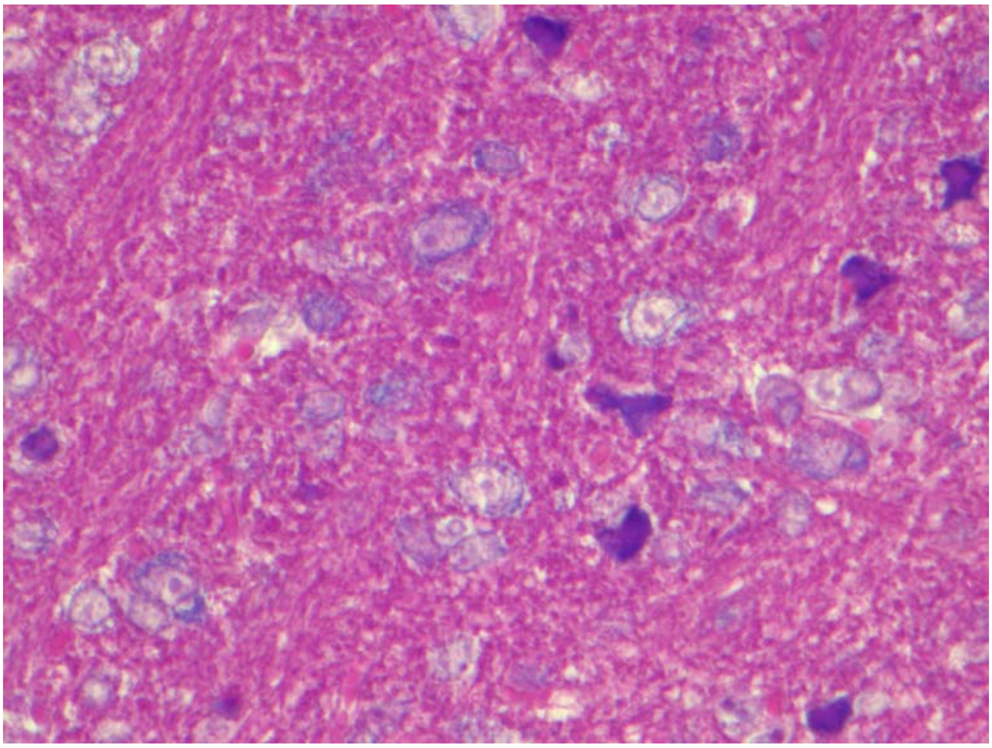

Supplement: Figure S4 — Seller's staining of brain sections from 8-week old mice. BALB/C mice were infected i.m. with 106 TCID50 of DUVV-NL07 (S4a) or RABV-PV (S4b) or SHBRV-18 (S4c). Seller's staining was performed as described in “Laboratory Techniques in rabies” Forth Edition, World Health Organization, Geneva 1996. (PDF) [file ppat.1002682.s004.pdf]
