# Supplementary material for: In Vitro and In Vivo Isolation and Characterization of Duvenhage Virus
Source: PLoS Pathog. 2012 May 24;8(5):e1002682. doi: 10.1371/journal.ppat.1002682 (PMC3359985; doi:10.1371/journal.ppat.1002682)
Supplement: Table S1 — Virus isolates and their respective accession numbers used to construct the phylogenetic tree depicted in Figure 1. (DOC) [file ppat.1002682.s005.doc]

Supplementary Table S1: Virus isolates and their respective accession numbers used to construct the phylogenetic tree depicted in Figure 1. 

Accession number	Lyssavirus isolate 
	
EU293116.1	Rabies virus isolate 9704ARG	
EU293115.1	Rabies virus isolate 9147FRA	
EU293113.1	Rabies virus isolate 9001FRA	
EU293111.1	Rabies virus isolate 8764THA	
EU293121.1	Rabies virus isolate 8743THA	
EU293110.1	Lagos bat virus isolate 8619NG	
EU293108.1	Lagos bat virus isolate 0406SE	
EU293118.1	Mokola virus isolate 86101RCA	
EU293117.1	Mokola virus isolate 86100CAM	
EU293120.1	Duvenhage virus isolate 94286S	
EU293119.1	Duvenhage virus isolate 86132S	
EU623444.1	Duvenhage virus isolate SA06	
JN986749	Duvenhage virus isolate NL07 P1	
EU293109.1	European bat lyssavirus 1 isolate	
EU293112.1	European bat lyssavirus 1 isolate	
EF157976.1	European bat lyssavirus 1 isolate	
NC_009527.1	European bat lyssavirus 1	
NC_009528.1	European bat lyssavirus 2	
EF157977.1	European bat lyssavirus 2 	
EU293114.1	European bat lyssavirus 2 	
AF418014.1	Australian bat lyssavirus	
AF081020.2	Australian bat lyssavirus	
NC_003243.1	Australian bat lyssavirus	
EF614261.1	Khujand lyssavirus	
EF614259.1	Aravan virus 	
GQ918139.1	Rabies virus strain CVS-11	
AY705373.1	Rabies virus strain SHBRV-18	
NC_001542.1	Rabies virus reference strain	
